# Options and Obstacles for Designing a Universal Influenza Vaccine

**DOI:** 10.3390/v6083159

**Published:** 2014-08-18

**Authors:** Yo Han Jang, Baik Lin Seong

**Affiliations:** 1 Department of Biotechnology, College of Life Science and Biotechnology, Yonsei University, 50 Yonsei-ro, Seodaemun-gu, Seoul 120-749, South Korea; E-Mail: yhjh0323@yonsei.ac.kr; 2 Translational Vaccine Research Center, Yonsei University, 50 Yonsei-ro, Seodaemun-gu, Seoul 120-749, South Korea; 3 Translational Research Center for Protein Function Control, Yonsei University, 50 Yonsei-ro, Seodaemun-gu, Seoul 120-749, South Korea

**Keywords:** influenza virus, universal vaccine, hemagglutinin, live vaccine, cross-protection

## Abstract

Since the discovery of antibodies specific to a highly conserved stalk region of the influenza virus hemagglutinin (HA), eliciting such antibodies has been considered the key to developing a universal influenza vaccine that confers broad-spectrum protection against various influenza subtypes. To achieve this goal, a prime/boost immunization strategy has been heralded to redirect host immune responses from the variable globular head domain to the conserved stalk domain of HA. While this approach has been successful in eliciting cross-reactive antibodies against the HA stalk domain, protective efficacy remains relatively poor due to the low immunogenicity of the domain, and the cross-reactivity was only within the same group, rather than among different groups. Additionally, concerns are raised on the possibility of vaccine-associated enhancement of viral infection and whether multiple boost immunization protocols would be considered practical from a clinical standpoint. Live attenuated vaccine hitherto remains unexplored, but is expected to serve as an alternative approach, considering its superior cross-reactivity. This review summarizes recent advancements in the HA stalk-based universal influenza vaccines, discusses the pros and cons of these approaches with respect to the potentially beneficial and harmful effects of neutralizing and non-neutralizing antibodies, and suggests future guidelines towards the design of a truly protective universal influenza vaccine.

## 1. Introduction

Influenza viruses belong to the family Orthomyxoviridae and contain a segmented RNA genome. Due to its high propensity for genetic mutations, especially in major surface antigens (hemagglutinin (HA) and neuraminidase (NA)), the virus can easily evade preexisting immunity acquired from previous exposure to the virus, thereby causing seasonal epidemics, with 3–5 million cases of severe illness and 250,000–500,000 deaths each year (antigenic drift) [1,2,3]. In addition, influenza viruses occasionally exchange their genetic materials and give rise to a new virus subtype. When a new HA and/or NA is introduced into a population that has little or no preexisting immunity to the new subtype virus (antigenic shift), morbidity and mortality rates become substantially higher than typical seasonal epidemics, as seen in the past influenza pandemics, such as the 1918 Spanish flu pandemic [4].

Vaccination has been considered the most cost-effective measure to prevent and mitigate influenza infections. Currently used seasonal influenza vaccines include the HA and/or NA antigens derived from the virus subtype that is most likely to circulate during the impending season. Such vaccines are considered to elicit neutralizing antibodies directed predominantly to antigenic sites in the variable globular head domain of HA that mediates the receptor binding and virus entry into the cells [5]. Such neutralizing activity, however, is generally strain-specific, because the variable globular head domain of the HA differs, even among the viruses within the same HA subtype, and therefore, seasonal influenza vaccines need to be updated almost annually to match the antigenicity of newly circulating viruses [6]. Occasionally, the antigenic mismatches between vaccine strains and circulating viruses lead to substantial decrease in vaccine efficacy [7,8]. More importantly, the emergence of pandemic viruses and zoonotic influenza outbreaks are highly unpredictable [9,10], especially with respect to the origins from which the pandemic viruses acquire its genetic sources and virulence factors.

Imperfections in the surveillance of circulating viruses and the limited breadth of the protection efficacy of HA globular head-based vaccines against heterologous influenza viruses have motivated the development of vaccines with broader and longer-lasting protection, ultimately a universal vaccine that would provide protection against diverse influenza virus strains regardless of their subtypes. Naturally, the regions of viral proteins that are highly conserved across viral strains became the main focus of attention, with the hope that eliciting antibody responses to such conserved regions would present as a promising way of developing universal influenza vaccines. For this purpose, conserved viral proteins or domains, including the M2 extracellular (M2e) domain and HA stalk domain, have been studied and were demonstrated to provide better cross-protection against diverse viruses than HA globular head-based vaccines [11,12].

While these ideas shed light on the feasibility of conserved domain-based universal influenza vaccines, several challenges remain to be solved: the lower immunogenicity of the conserved regions compared to the variable globular head domain, the incomplete protective efficacy against heterosubtypic strains and the vaccine-induced enhancement of viral diseases [13]. This paper mainly focuses on more recent achievements in HA stalk-based universal influenza vaccines, discusses the pros and cons of these approaches with respect to the beneficial and harmful effects and ends with suggestions for future guidelines towards the design of truly protective universal influenza vaccines.

## 2. Antigenicity and Structure of HA

**Figure 1:**
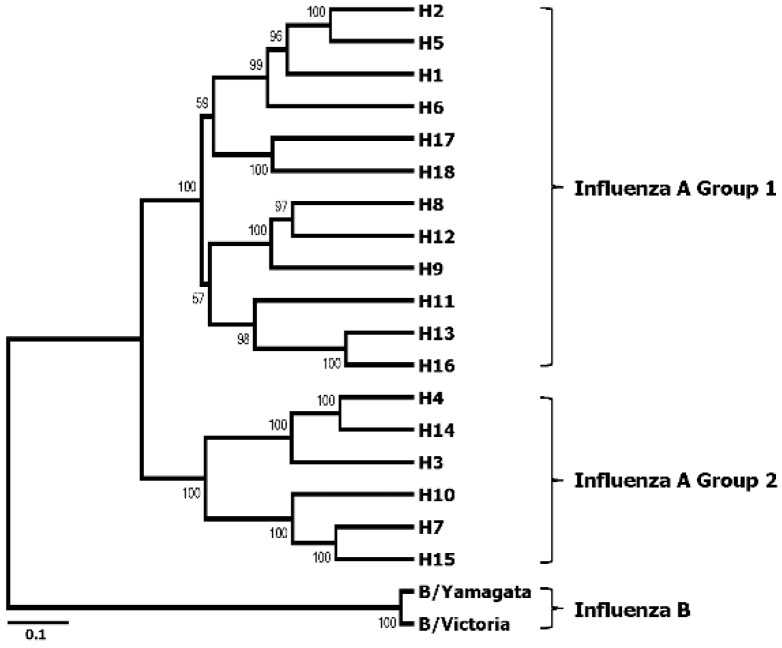
Phylogenetic tree of all subtypes of hemagglutinin (HA) of influenza A and influenza B viruses. The rooted phylogenetic tree was generated based on a full-length amino acid sequence comparison among influenza A and B viruses. The representative sequence of each HA subtype, including recently added H18, was obtained from the Influenza Virus Resource of NCBI for multiple alignments, and the phylogenetic tree was generated by the ClustalW algorithm in Mega version 6.0 using UPGMA method. The scale bar represents a 10% amino acid change, and the bootstrap values are given at each node.

**Figure 2:**
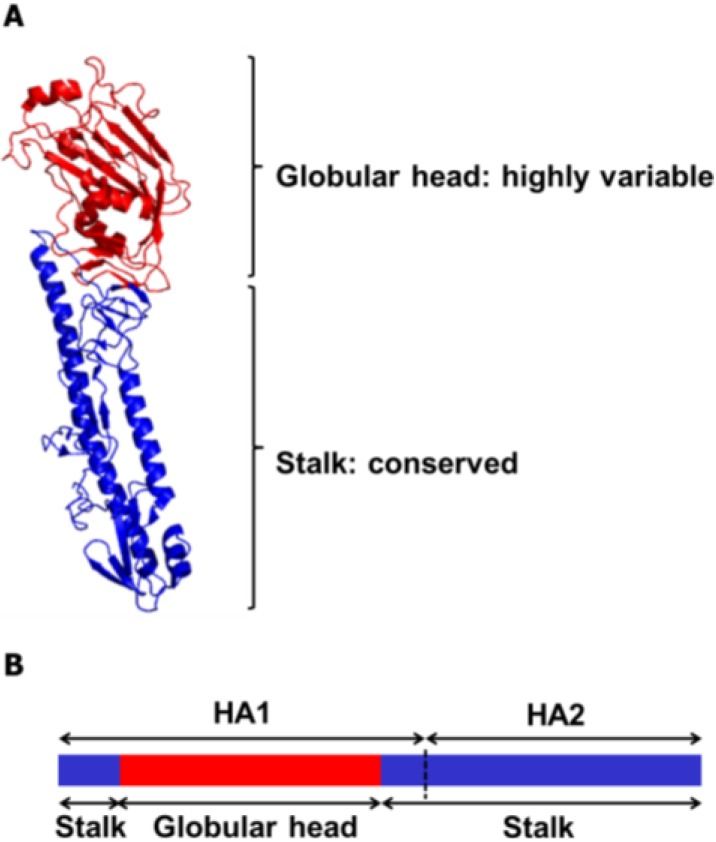
Ribbon diagram and schematic representation of the influenza HA protein. (**A**) Each monomeric HA is comprised of two functional domains. The highly variable globular head domain (red) contains a receptor binding site and major antigenic epitopes. The stalk domain (blue) is located in membrane proximal region and shows a high degree of conservancy among influenza viruses. The HA structure was downloaded from the Protein Data Bank (HA of A/swine/Iowa/15/1930 (H1N1), PDB ID 1RUY), and the final image was produced by the PyMOL program). (**B**) The globular head domain is entirely formed by the HA1 subunit (Residues 55–271 in H3 numbering), whereas the stalk domain is formed by the rest of the HA1 subunit and the entire HA2 subunit.

HA is a homotrimeric glycoprotein that mediates influenza viral entry via cellular attachment and membrane fusion events [14,15]. To date, 18 subtypes of HA (H1~H18) of the influenza A viruses have been identified [16], which are classified into two phylogenetic groups according to their amino acid sequence homologies. Likewise, influenza B viruses are classified as either Victoria lineage or Yamagata lineage [17,18] (Figure 1). During the infection cycle of the virus, each HA precursor (HA0) is processed by host proteases into two subunits, HA1 and HA2, which remain cross-linked via a disulfide bond. The HA comprises two domains, the globular head domain and the stalk domain, functionally and antigenically distinct from each other (Figure 2). The former is formed entirely from the HA1 and contains the receptor binding site and five antigenic sites, while the latter is formed from both the rest of HA1 and all of HA2 and is located proximal to the membrane region [19,20]. The globular head domain mediates the attachment of the virus to the target cells, and antibodies directed to this domain block receptor binding of the HA and, thereby, inhibit viral entry, demonstrating hemagglutinin inhibition (HI) activity and viral neutralization (VN) activity. However, the globular head domain is highly variable across viruses and tends to change under immune pressure and, hence, easily evades the neutralizing antibodies induced by previous vaccinations or infections. On the other hand, the stalk domain of the HA has the essential role of the fusion of the viral and endosomal membranes and subsequent release of the viral genome into the cytoplasm. This domain has been shown to remain relatively well conserved across viruses, but is far less immunogenic than the bulky globular head domain [21]. Thus, antibodies directed to this domain occur only at a low frequency [22,23], which is why stalk domain-specific neutralizing antibodies were not discovered until early in the 1990s [24].

## 3. Broadly Neutralizing Monoclonal Antibodies Specific to HA

The first report describing the presence of broadly neutralizing antibodies specific to the HA stalk domain demonstrated that the murine monoclonal antibody C179 recognized the HA stalk domain, thereby inhibiting membrane fusion and neutralizing both H1 and H2 subtypes, even without the HI activity [24]. Subsequently, a series of studies have identified human broadly neutralizing antibodies (bnAbs) targeting this domain, offering exciting prospects for the design of a universal influenza vaccine that protects against many influenza virus subtypes. Firstly, A06 antibody identified from the human library was derived from a survivor infected with highly pathogenic H5N1 infection neutralized H5N1, seasonal H1N1 and 2009 pdmH1N1 viruses, and its binding epitope was predicted to be the HA stalk region [25,26] (Table 1). In addition, the panel of bnAbs, including CR6261, was identified via screening of the human antibody phage-display library [27]. These antibodies showed broad heterosubtypic neutralizing activity within antigenically diverse Group 1 influenza subtypes, including H1, H2, H5, H6, H8 and H9. The binding regions were suggested to be on the HA stalk domain initially by 3D modeling and binding analyses, which was later confirmed via the crystal structures of the CR6261 in complex with HA [19]. Another group also identified human bnAbs that were effective against all Group 1 influenza viruses tested, including the 1918 Spanish flu H1 and avian H5 subtypes, and the structural analysis of the monoclonal antibody F10-HA stalk domain complex suggested that those antibodies share a common epitope in the HA stalk domain, inhibiting membrane fusion rather than receptor binding [28]. However, such bnAbs were shown to be effective against Group 1 influenza subtypes, but not Group 2 subtypes, such as H3 and H7 viruses [27], probably due to the low sequence identity between the stalk domains from the two groups [29,30]. Subsequently, Group 2-specific bnAbs, CR8020 and CR8043, were identified and were shown to bind to the HA stalk domain, inhibiting the conformational rearrangement of the HA [31,32], leading to the suggestion that a cocktail of two bnAbs specific to Group 1 and Group 2 influenza subtypes could confer protection against almost all influenza A viruses. Moreover, a new bnAb FI6v3 was isolated from human plasma cells that recognized all 16 HA subtypes and neutralized both Group 1 and 2 influenza viruses through binding to the HA stalk domain [33] (Table 1). While the FI6v3 share the same mechanism of viral neutralization with the Group 1- or 2-specific bnAbs, the crystal structure of the FI6v3-HA complex revealed that FI6v3 could accommodate the structural differences between Group 1 and 2 HA stalk domains. This involves a group-distinctive microenvironment and the orientation of the Trp^21^ residue by contacting a larger area using both antibody V_H_ and V_K_ chains, in contrast with the Group 1- or 2-specific bnAbs that use only the V_H_ chain. The results will be conductive to the design of a pan-influenza A universal vaccine by eliciting such antibodies. A highly conserved epitope across all 16 influenza A subtypes and influenza B viruses and its corresponding bnAb CR9114 were also discovered, and its prophylactic efficacy against influenza A and B viruses was evaluated in mice [34]. The CR9114 was shown to bind the HA stalk domains of both influenza A viruses and influenza B viruses, but neutralized only influenza A viruses in an *in vitro* assay. However, the passive transfer of the antibody protected the mice from lethal challenges with influenza A and B viruses (Table 1), implying that the protection *in vivo* against the influenza B viruses by the CR9114 depended heavily on antibody effector functions, such as antibody-dependent cell-mediated cytotoxicity (ADCC) or complement-dependent cytotoxicity (CDC), similar to that shown in M2e-based vaccines [35,36,37,38]. The hypothesis that the protection by the bnAbs may depend on both blocking membrane fusion by antibodies and antibody effector functions was supported by a recent study showing that bnAbs targeting the HA stalk domain requires the interactions between the antibody Fc domain and the Fc receptor on the cellular membrane for exerting their maximum neutralization activity *in vivo* [39]. In addition to the HA stalk domain, the globular head domain of HA has also been shown to contain conserved epitopes across influenza viruses, where their responsive monoclonal antibodies, such as CH65, 5J8, CR8033 and C05, confer broadly neutralizing activity by binding close to the receptor binding site of HA, interfering with viral attachment to cellular receptors [34,40,41,42] (Table 1).

These studies collectively demonstrate that antibodies directed to the conserved regions of HA occur in those who have been exposed to the viruses and that such antibodies confer broadly neutralizing activity against different subtypes of influenza viruses by inhibiting key functions of the HA, such as receptor binding or membrane fusion. The identification and characterization of these bnAbs specific to the HA of influenza A and B viruses not only present therapeutic and prophylactic strategies based on using such monoclonal antibodies, but also provide a basis for the development of a universal influenza vaccine that elicits such antibody responses.

**Table 1:** Selected human broadly neutralizing monoclonal antibodies specific to HA.

		Protection Specificity ^a^			
		Influenza A		Influenza B	
Monoclonoal Antibody	Binding Target	Group 1	Group 2		Ref.
A06	Stalk	H1, H5	NA ^b^	NT ^c^	[25,26]
CR6261	Stalk	H1, H2, H5, H6, H8, H9	NA	NT	[27]
F10	Stalk	H1, H2, H5, H6, H8, H9	NA	NT	[28]
CR8020	Stalk	NA	H3, H7	NT	[31]
CR8043	Stalk	NT	H3, H7, H10	NT	[32]
FI6v3	Stalk	H1, H5	H3, H7	NT	[33]
CR9114	Stalk	H1	H3	Yam, Vic	[34]
CR8033	Head	NT	NT	Yam, Vic	[34]
CR8071	Head	NT	NT	Yam, Vic	[34]
CH65	Head	H1	NT	NT	[40]
5J8	Head	H1	NT	NT	[41]
C05	Head	H1, H2, H9, H12	H3	NT	[42]

^a^ The protection specificity of each monoclonal antibody is based on viral neutralizing activity or hemagglutinin-inhibition activity *in* *vitro* or the protection ability for *in* *vivo* animal models presented in each reference. Note that the binding activity of each antibody for *in* *vitro* assay is not considered as protective activity in this review, and refer to indicated references for more information on the range of binding specificity to other HA subtypes. ^b^ NA indicates no neutralizing activity*.* ^c^ NT indicates not tested*.*

## 4. Eliciting Broadly Neutralizing Antibodies by Vaccination or Infection

Many studies described above have proven the occurrence of bnAbs directed to the conserved HA stalk domain and verified the prophylactic and therapeutic efficacies of such antibodies in passive transfer experiments in animal models [26,27,28,31,32,33,34]. However, it has been difficult to elicit sufficient levels of the bnAbs that confer the desired level of protection against diverse influenza viruses by current seasonal influenza vaccinations, which leaves most of the population with prior exposures to the viruses or with vaccination vulnerable to infection by heterologous viruses [22]. This has led to several studies for strategic variations of vaccination to boost the stalk-specific antibody responses. Priming with plasmid DNA encoding H1 HA followed by boosting with a seasonal trivalent vaccine or a replication-defective adenovirus vector encoding the same HA stimulated the production of broadly neutralizing antibodies against heterologous H1 viruses and other Group 1 H2N2 and H5N1 viruses [43]. In this study, the heterosubtypic neutralizing activity of the immune sera from immunized mice substantially diminished when the antibodies specific to the HA stalk domain were depleted by prior incubation with the stalk proteins, indicating that the protection depended primarily on the bnAbs induced by the prime/boost vaccination. Subsequent studies by the same group have revealed that previous exposures to the influenza viruses, either by infections or vaccinations, do not prevent the generation of the stalk-directed bnAbs, alleviating the concern that the bnAbs might be difficult to induce in humans with previous exposures to the viruses [44]. In support of this, a longitudinal analysis with human serum samples gathered over a 20-year period has revealed that the HA stalk-specific antibody titers increase over time, suggesting that boosts of the HA stalk antibodies could be achieved in humans with complex and varied previous exposure histories [45].

It has been shown that 2009 pdmH1N1 virus infections or vaccinations in humans preferentially induce antibodies with broad specificity to various influenza subtypes, many of which are directed to the HA stalk domain [46,47]. Consistent with this observation, HA stalk-reactive antibodies are efficiently boosted after sequential infections, initially with the seasonal influenza virus, followed by the 2009 pdmH1N1 virus. Moreover, the boosting effect was stronger with the 2009 pdmH1N1 infection than with a closely related drifted seasonal influenza virus [48]. It has also been suggested that in humans, infection with the 2009 pdmH1N1 virus containing HA proteins with a globular head domain that differs substantially from typical seasonal influenza viruses resulted in a boost in broadly neutralizing antibodies specific to the HA stalk domain [49]. Similarly, sequential influenza virus infections with two different H3N2 strains induced broadly reactive stalk antibodies, both in humans and mice [50]. Collectively, these clinical and preclinical data suggest that bnAbs specific to the HA stalk domain can be generated either by prime/boost vaccinations or sequential infections and that such a boosting effect becomes more robust by a second exposure to the HA containing the heterologous globular domain and the homologous stalk domain relative to the previous HA proteins.

## 5. Chimeric HA as Universal Influenza Vaccines

**Figure 3:**
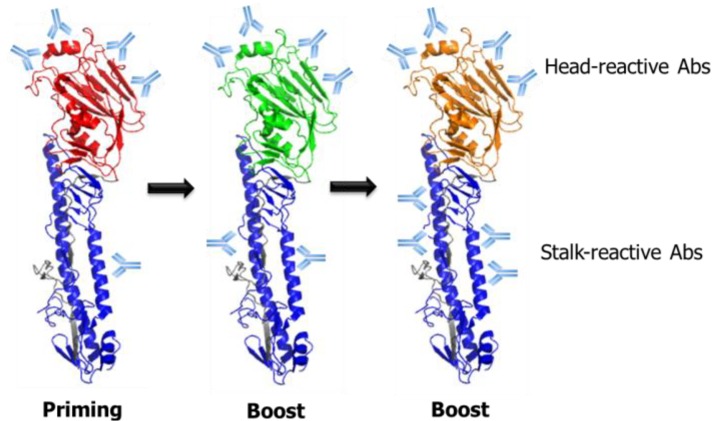
Schematic illustration of prime/boost vaccination strategy based on chimeric HA (cHA) for the boosting of HA stalk-reactive antibodies. With each priming or boost immunization, with the cHA comprising divergent globular head domains and the same stalk domains, the antibody responses are likely to be redirected toward the conserved stalk domain. This strategy has been applied to various vaccine formats, such as recombinant protein vaccines, DNA vaccines and virus-vectored vaccines. In addition, a similar approach could be potentially extended to live attenuated influenza vaccines based on the reverse genetic platform for engineered influenza viruses [57].

The findings that antibody responses toward the conserved HA stalk domain can be increased through prime/boost vaccinations provide a basis for the development of a universal influenza vaccine based on recombinant chimeric HA proteins that are comprised of the globular head domain and the stalk domain derived from different influenza subtypes (Figure 3). Priming by DNA encoding HA or a low dose infection followed by repeated immunizations with chimeric HA proteins containing the same stalk domain, but irrelevant head domain, stimulated stalk-directed polyclonal antibody responses and protected the immunized animals from lethal infections with heterologous influenza viruses [51,52,53,54]. Furthermore, passive transfer or CD8+ T-cell depletion experiments showed that the protection was mediated mainly by neutralizing antibodies against the stalk domain, demonstrating the feasibility of a chimeric HA-based universal vaccine strategy. While the studies described above all used the recombinant HA proteins produced through a baculovirus expression system, recent studies have reported the production of influenza HA proteins using *Escherichia coli* expression systems, although their vaccine efficacy remains to be elucidated [55,56]. High yield production of the HA proteins in an *E. coli* system would be usefully implemented in the production of recombinant universal influenza vaccines, as well as in the evaluation of HA-specific antibody responses induced by the vaccines. Alternatively, viral vector-based delivery of the chimeric HAs using engineered influenza A or B viruses, vesicular stomatitis virus and adenovirus is also able to stimulate the HA stalk-specific antibody responses and provided broad protection against heterologous influenza infections in mice and ferrets [51,52,54], suggesting a wide range of options for the HA stalk-based universal vaccine strategies.

## 6. Guiding Antibody Responses toward the HA Stalk Domain by Modulation of the Glycosylation State

There has been convincing evidence that glycosylations in the HA globular head domain modulate the virulence and antigenic properties of influenza viruses. During evolution in humans, influenza A viruses have accumulated genetic mutations at the antigenic sites of HA globular head domain and resulted in changes of the glycosylation pattern, which was shown to be associated with immune evasion or changes in viral antigenicity [58,59]. More specifically, it has been demonstrated that the glycosylation state, in particular, residues in the HA globular head domain, affecting not only viral pathogenesis, but also the breadth of cross-reactive antibody responses to the viruses [60,61,62]. These data and others generally suggest that immune responses against an immunodominant region of the HA is likely to be shielded by introducing additional glycosylation, causing antigenic focus to be redirected toward other epitopes unmasked by the glycosylation. This speculation was supported by a recent study demonstrating that, over time, mutations in the HA globular head domain are focused on antigenic regions that are unmasked by glycosylation [63]. Those findings provided a rationale to designing a universal influenza vaccine that expresses HA proteins with hyperglycosylation in their globular head domain. Another study has demonstrated that mice immunized with hyperglycosylated HA proteins carrying artificially introduced seven N-linked glycosylation sites in their globular head domain developed enhanced stalk-directed antibody responses while dampening head-reactive responses. These mice also showed better protection against morbidity and mortality upon lethal infection than mice that received wild-type HA proteins [64]. Although the protective efficacy of this approach against heterologous infections has not been extensively examined yet, the broad reactivity of the stalk-directed antibody responses in *in vitro* assays suggests that shielding immunodominant epitopes in the HA globular head domain by hyperglycosylation could be applied to increase the stalk-reactive antibody responses.

## 7. Hurdles in HA Stalk-Based Universal Influenza Vaccine Development

### 7.1. Vaccine-Induced Enhancement of Viral Infections

Although vaccination has been considered the most effective measure against infectious diseases, there have been some reports of rare vaccine-induced enhancement of susceptibility to virus infection or of aberrant viral pathogenesis in humans and animals. Such phenomena were shown to render vaccine-recipients more vulnerable to subsequent viral infection than non-vaccinated subjects, rather than protecting them. Representative examples of the vaccine-induced enhancement of viral infection have been documented in the lentivirus family, including the human immunodeficiency virus (HIV), simian immunodeficiency virus (SIV) and feline immunodeficiency virus (FIV) [65,66], and were also observed in other families, such as Flaviviridae, Coronaviridae and Paramyxoviridae [67]. Similarly, vaccine-associated enhanced respiratory disease (VAERD) has been reported in multiple respiratory infections, including respiratory syncytial virus (RSV) and atypical measles [68,69,70]. Although several potential mechanisms underlying the vaccine-induced enhancement of viral infection were suggested, the antibody-dependent enhancement (ADE) is believed to play a major role through prolonged contact of the virus/antibody complex with the antibody Fc receptor (FcR) on the cell membrane, thereby increasing the chance of viral entry into the cells [67]. Thus, balancing between the induction of protective immunity and the induction of aberrant viral pathogenesis should be of serious consideration in evaluating vaccine safety.

Unfortunately, the VAERD has also been reported in some cases of mismatched influenza vaccine and challenge viruses, especially in swine. Inactivated vaccines of classical swine H1 virus provided protection in pigs against homologous challenges, but failed to protect the animals from challenge with homosubtypic, but heterologous, H1 viruses and enhanced the severity of pneumonia compared to the non-vaccinated control group [71]. In the following studies, immunization with inactivated H1 vaccines did not protect immunized pigs against the heterologous 2009 pandemic H1N1 virus challenge, but resulted in enhanced illness of pneumonia and a significantly elevated proinflammatory cytokine profile in the lungs [72,73]. Subsequently, a recent study has provided more detailed insights into the effects of vaccination-induced antibody responses on the VAERD observed in the previous studies [74]. In this study, the anti-HA antibodies induced in pigs immunized with an inactivated H1N2 influenza vaccine failed to show neutralizing activities against the heterologous 2009 pdmH1N1 virus, but only bound to the HA stalk domain. Interestingly, it was also shown that anti-HA stalk antibodies, in sharp contrast to previously identified bnAbs with inhibitory effects on membrane fusion, had membrane fusion-enhancing activity in cell culture, which was correlated with increased lung pathology after infection by the 2009 pdmH1N1. Of note, although the H1N2 vaccine-induced antibodies were cross-reactive to the HA stalk domain of the infecting 2009 pdmH1N1 virus, they compromised the neutralizing activity of globular head-reactive neutralizing antibodies, especially when the latter were present at low titers. Far from generalization, however, it could be suggested that there is a delicate balance between the levels of neutralizing antibodies targeting the globular domain and non-neutralizing fusion-enhancing HA stalk-specific antibodies, which is likely to determine the clinical outcome of mismatched influenza infections [74]. One caveat to this interpretation is the likely disparity between antibody responses induced by the prime/boost vaccination or recombinant chimeric HA strategies described above and those in the swine model studies, especially with respect to the titers or affinity of the antibodies induced. This mechanism underlying the influenza-related VAERD elucidated in the swine model may present one explanation for the VAERD-like phenomenon in several observational studies, in which unexpectedly enhanced illness was observed in the 2009 pdmH1N1-infected humans with the prior receipt of the 2008–09 seasonal trivalent inactivated influenza vaccine [75,76,77]. However, it remains unclear whether the VAERD in swine and a similar phenomenon in humans are unique to the 2009 pdmH1N1 infection. In addition, it should be noted that there are many clinical reports of a small, but apparent, protective effect of the inactivated or live attenuated seasonal influenza vaccines against pandemic virus infection in humans, despite poor cross-reactive serologic responses [78,79,80]. Furthermore, it has been reported that the M2e-based DNA vaccine could also develop a similar VAERD-like illness in vaccinated pigs [81], and that adenoviral vectored HA vaccine did not induce the VAERD in pigs, while adjuvanted whole inactivated vaccine did [82], rendering it more complicated to identify the responsible factors or situations for such a phenomenon. Thus, it is increasingly important to understand how previous vaccinations or infections shape the immune responses against heterologous influenza infections. We suggest that the VAERD should be carefully monitored when devising a universal influenza vaccine that targets conserved epitopes, such as the HA stalk domain.

### 7.2. Weak Protective Efficacy of HA Stalk-Based Vaccines

Although it has been conclusively proven that HA stalk-reactive antibodies are able to neutralize heterologous influenza viruses and can be boosted by repetitive immunizations with a chimeric HA or a hyperglycosylated HA vaccine, their protective efficacy tends to be less potent than conventional globular head-based vaccines. Despite multiple immunizations with those vaccines, immunized animals showed mild clinical symptoms of varying degrees of weight loss and high titers of viruses in their respiratory tracts after lethal challenges with heterologous influenza viruses [52,53,54,64], implying that current HA stalk-based vaccines provided only partial protection, especially against more pathogenic strains, such as the 2009 pdmH1N1 or recent H7N9 viruses. It should also be mentioned, however, that even the standard of care—a single vaccination of non-adjuvanted split vaccines—does not completely protect mice from challenge either; even more so for ferrets. From a practical standpoint, therefore, the HA stalk-based vaccines could be beneficial in improving broad-spectrum protection, especially in a situation of vaccine mismatch, in which the current inactivated or split vaccines provides little protection against heterologous influenza viruses.

The lesser potency of the HA stalk-reactive antibodies could be reasonably attributed to their intrinsic nature. HA stalk-specific antibodies were shown to not only prevent the proteolytic cleavage of the HA0 precursor into HA1 and HA2, but also to stabilize the pre-fusion state of the protein, thereby inhibiting the subsequent fusion process that is essential for the release of viral genome into the cytoplasm for successful infection [31,34,83]. However, they do not prevent viral entry into the cells, in contrast to the head-specific antibodies that block receptor binding and the endocytosis of the virus, as well as the release of progeny virus particles [34,83]. Naturally, viruses inside the cell have a better chance to initiate the replication cycles than those trapped outside the cell. Another possible explanation for the weak protective efficacy is the poor accessibility of the bnAbs to the HA stalk domain on the virion. This question has been difficult to answer, because our present knowledge of the HA structure is derived largely from crystallographic analyses of soluble ectodomains of trimeric HA or electron microscopic analyses of chemically-stained HA trimers [34,84], which may not accurately reflect the antibody accessibility to a naive HA trimer displayed on intact whole virion. Recently, cryo-electron tomography of the HA on an intact 2009 pdmH1N1 virus has become available, suggesting that, despite their close packing on the viral membrane, ~75% of HA trimers on intact virions can be bound with stalk-specific antibodies [85], showing the feasibility of an HA stalk-based universal influenza vaccine. It should also be noted that the degree of homology in the HA stalk domains also varies with the antigenic distance between different influenza viruses (Figure 1). The protective efficacy of HA stalk-reactive bnAbs is therefore likely to diminish against antigenically distant viruses, due to the low binding strength between the antibodies and antigens, as reflected by the poor protection of Group 1 HA stalk-based recombinant HA vaccine against Group 2 H3N2 virus infection [51]. To date, there is no data addressing the breadth of protection of the universal influenza vaccine constructs against a full range of Group 1 or Group 2 influenza virus subtypes. Provided the unpredictability of a sudden emergence of an influenza outbreak, it would be worthwhile to evaluate the protective spectrum of various “universal” influenza vaccine constructs.

As demonstrated in the swine model, VAERD is closely related with the HA stalk-reactive antibodies as infectivity-enhancing factors that support viral entry into the cells and compromises the neutralizing effect of head-reactive antibodies [74]. Although the prime/boost regimen with a chimeric or hyperglycosylated HA has been shown to induce broadly protective immunity against heterologous influenza infections, it remains to be further explained if the polyclonal antisera induced by these vaccine constructs produce mixtures of infectivity-inhibitory antibodies and infectivity-enhancing antibodies. Alternatively, there remains a possibility of a threshold of stalk-reactive antibody titer or affinity that determines the fate of a clinical outcome by viral infections. These hypotheses are supported by several previous reports of the antibody-dependent enhancement of viral infection in various virus families, such as the dengue virus and HIV [86,87,88,89,90].

Considering the relatively weak immunogenicity of the HA stalk domain and, hence, low frequencies of the responsive antibodies, special caution should be given to the potential infectivity-enhancing effect, which could become pronounced against antigenically distant influenza viruses, consequently weakening the protective potency of vaccination. Given that the 2009 pandemic H1N1 infection or vaccination in humans could induce the HA stalk-reactive broadly neutralizing monoclonal antibodies that were as effective as the globular head-reactive antibodies [46,91], the remaining challenge is to design influenza vaccines or vaccination strategies that can generate sufficiently high levels of such antibodies to confer complete protection against the multiple influenza virus strains without demonstrating any harmful effects.

## 8. Options for Improving the Potency of Universal Influenza Vaccines

### 8.1. Epitope-Focused Vaccine Design

Recently, one research group has reported an epitope-focused vaccine design strategy, in which the continuous or discontinuous viral epitopes of RSV or HIV were transplanted into biologically stable scaffold proteins. The resulting epitope-scaffold immunogen complexes, either in monomeric proteins or multivalent virus-like particle (VLP) particles, were able to induce potent neutralizing antibodies in mice and nonhuman primates [92,93,94,95,96]. Furthermore, in a computational approach using a robust molecular modeling platform, it was possible to tailor the scaffold structures for particular epitopes to accurately mimic the native structure [96]. Since this strategy allows immune responses to focus on the selected epitopes of interest, it would be possible to guide antibody responses toward the less immunogenic, but conserved epitopes to elicit broad antiviral immunity in a variety of other vaccine targets. As the broadly neutralizing monoclonal antibodies have defined various target epitopes in the influenza HA globular head and stalk domain, this epitope-focused vaccine design provides a powerful universal vaccine platform for substantially increasing the cross-reactive antibody responses to those conserved epitopes. For example, a scaffold conjugated with the HA stalk epitope that was shown to be conserved across both influenza A and B viruses [34] might elicit a pan-influenza neutralizing response in a more focused manner than a vaccine construct that expresses both the globular head domain and stalk domain. However, a similar approach has shown that VLPs carrying multiple copies of a broadly neutralizing epitope recognized by the CR6261 or F10 antibody were poorly immunogenic in mice and did not provide protection against lethal challenge with heterologous virus [97], underscoring a judicious choice for epitopes, scaffold proteins and carrier VLPs to focus the immune responses toward the target epitopes.

### 8.2. Live Attenuated Influenza Vaccine as a Potential Platform for Universal Influenza Vaccine

Despite the recent advances in universal influenza vaccines described above, live attenuated influenza vaccines (LAIVs) remain unexplored. This is possibly due to the relatively low efficiency of the LAIVs in inducing systemic anti-HA antibody responses and a lack of precise immune correlates of protection provided by the LAIVs [98,99,100]. However, a wealth of clinical and experimental evidence has clearly shown that cold-adapted LAIVs (CAIVs) are capable of inducing cross-protective immune responses encompassing both cellular immunity and humoral immunity [101,102,103]. In particular, it has been shown that CAIVs against the 2009 pdmH1N1 virus conferred broad protection against antigenically distant seasonal and avian H5 influenza A viruses [104,105]. One key question to address with respect to the CAIV-based universal influenza vaccine is how efficiently it enables the induction of HA stalk-reactive antibodies. In a previous study, low doses (10^3^ and 10^4^ plaque forming units) of a single immunization with a CAIV or the NS1-truncated virus expressing A/Puerto Rico/8/34 (H1N1) HA did not induce detectable stalk-reactive antibodies in mice, whereas the same infection doses of a wild-type virus could [48]. The results could be explained by the lower replication level of the attenuated virus than the wild-type virus. Therefore, it is possible that multiple vaccinations with higher infection doses of a CAIV may be able to induce stalk-reactive antibodies as efficiently as the recombinant HA vaccines. While the LAIVs have been considered to be poorly immunogenic in inducing serum antibody responses, recent studies have clearly demonstrated that such low levels of initial responses by the LAIVs can be expanded rapidly upon subsequent boosting with other vaccine formats. For instance, priming with H5N1 LAIV followed by boosting with an inactivated or subvirion vaccine resulted in high and broad cross-clade immunogenicity against multiple H5N1 viruses in animals and humans [106,107]. Although these studies did not specifically measure the HA stalk-specific antibody responses, the results suggest that the LAIVs could also be included in the prime/boost vaccination for a universal vaccine strategy.

Given that stalk-reactive antibodies can be boosted more efficiently when the HAs expressed by two viruses have different head domains [47,48], various combinations of CAIVs carrying different HA subtypes could be tested for their efficiency in inducing stalk-reactive antibodies and protective efficacy against heterologous influenza viruses (Figure 3). A possible benefit of a CAIV-based universal influenza vaccine is the collective delivery of intact surface antigens, including HA, NA and M2. It has been reported that the NAs of influenza A and B viruses also carry conserved regions or immunogenic epitopes [108,109,110], making them attractive targets for the development of a universal influenza vaccine. Furthermore, there is a report showing that LAIVs containing truncated NS1 did not induce VAERD, but provided protection against heterologous influenza infection in the swine model [111], alleviating the concern for VAERD induced by inactivated influenza vaccines [74]. Parallel to the potential advantages when devising the universal influenza vaccines, the LAIVs are relatively intolerant to genetic modifications that compromise the viability of a vaccine strain, which may limit the options for genetic approaches to induce the HA stalk-reactive antibodies. On the other hand, it has been demonstrated that moderate changes to the influenza viral genome have little effect on the antigenicity and productivity of the live vaccines, as exemplified by several studies involving NS1-truncation, the modification of HA cleavage site or the introduction of caspase recognition motifs into the viral proteins [112,113,114]. Thus, further studies on rational genetic modifications in the HA that do not affect the viral viability and antigenicity coupled with novel vaccination strategies to preferentially induce HA stalk-specific antibodies would turn LAIVs into ideal targets for a more protective and safe universal influenza vaccine.

## 9. Conclusions

In the last decade, we have witnessed remarkable achievements in controlling influenza virus infections by vaccinations. The highly conserved HA stalk domain of the viruses emerges as a hopeful target for vaccines that would confer broadly protective immunity against various influenza subtypes. Many studies have identified a panel of broadly neutralizing antibodies with specificity to this domain, which provided exciting prospects of a rational design of a universal influenza vaccine that elicits such antibody responses. Along with successful results in broadening protection coverage against diverse influenza viruses, challenging issues still persist, such as incomplete protective efficacy, potentially vaccine-induced enhancement of viral infections and limited protection against different groups of influenza A viruses and influenza B viruses. A better understanding of antigen processing and thepresentation mechanisms of the host immune system, as well as the counteractive evasion strategies of the influenza virus will ultimately guide us toward a truly universal influenza vaccine.
